# Perioperative Patient-Initiated Communication in Gender-Affirming Mastectomy

**DOI:** 10.3390/jcm13123368

**Published:** 2024-06-07

**Authors:** Christian X. Lava, Isabel A. Snee, Karen R. Li, George L. O’Hara, Niyati P. Bhatt, Oscar J. Manrique, Kenneth L. Fan, Gabriel A. Del Corral

**Affiliations:** 1 Georgetown University School of Medicine, Washington, DC 20007, USA; cl1449@georgetown.edu (C.X.L.); is455@georgetown.edu (I.A.S.); krl67@georgetown.edu (K.R.L.); glo6@georgetown.edu (G.L.O.); nb826@georgetown.edu (N.P.B.); 2 Department of Plastic and Reconstructive Surgery, MedStar Georgetown University Hospital, Washington, DC 20007, USA; kenneth.l.fan@medstar.net; 3 Department of Plastic and Reconstructive Surgery, University of Rochester Medical Center, New York, NY 14627, USA; oscar.j.manrique@gmail.com; 4 Department of Plastic and Reconstructive Surgery, MedStar Franklin Square Medical Center, Baltimore, MD 21237, USA

**Keywords:** transgender, mastectomy, patient-initiated communication, quality improvement

## Abstract

**Background**: Gender-affirming mastectomy (GAM) improves the psychosocial functioning and quality of life of transgender and non-binary (TGNB) individuals. However, the perioperative period is often marked by emotional stress, concerns about surgical outcomes, and physical discomfort. While inpatient procedures provide multiple opportunities to engage with and educate patients, outpatient surgeries, such as GAM, pose a unique challenge as patients are followed for <24 h postoperatively. Given the heightened emotional and psychological distress related to gender dysphoria TGNB individuals often experience, addressing these gaps can significantly improve outcomes. This study aims to characterize patient and surgical characteristics associated with patient-initiated communication (PIC) frequency in this population. **Methods**: A single-center retrospective review of TGNB patients undergoing GAM from February 2018 to November 2022 was conducted. Demographics, surgical characteristics, and frequency of and reasons for perioperative PIC (30 days before and after surgery) were recorded. The primary outcome was the incidence of perioperative PIC. The secondary outcomes included (1) the rationale for PIC and (2) patient and surgical characteristics associated with PIC. **Results**: A total of 352 patients were included. Of these, 285 (74.6%) initiated communication in the perioperative period, totaling 659 PICs. The median age was 25.0 (interquartile range [IQR]: 9.0) years. The median body mass index (BMI) was 28.5 (IQR: 8.5) kg/m^2^. The mean number of PICs was 0.7 ± 1.3 preoperatively and 1.3 ± 1.7 postoperatively (*p* < 0.001). The most frequent preoperative PIC subjects were administrative issues (AI; n = 66, 30.7%), preoperative requirements (n = 43, 20.0%), and cost and insurance (n = 33, 15.0%). The most frequent postoperative PIC subjects were wound care (n = 77, 17.3%), AI (n = 70, 15.0%), activity restrictions (n = 60, 13.5%), drainage (n = 56, 12.6%), and swelling (n = 37, 8.3%). Collectively, older patients (β = 0.234, *p* = 0.001), those with a history of major depressive disorder or generalized anxiety disorder (2.4 ± 3.0 vs. 1.7 ± 1.9; *p* = 0.019), and those without postoperative drains (n = 16/17, 94.1% vs. n = 236/334, 70.7%; *p* = 0.025) engaged in higher levels of PIC. There were no significant associations between other patient characteristics, perioperative details, or complications and PIC frequency. **Conclusions**: Perioperative PIC is prevalent among the majority of GAM patients at our institution, with age, psychiatric diagnosis, and postoperative drain use identified as significant predictors. To mitigate PIC frequency, it is crucial to ensure adequate support staffing and provide comprehensive postoperative instructions, particularly concerning activity restrictions and drainage management. These interventions may reduce PICs in high-volume centers. Further research should investigate targeted interventions to further support TGNB patients during the perioperative period.

## 1. Introduction

The number of gender-affirming chest surgeries, often referred to as “top surgery”, performed in the United States increased from 2700 in 2016 to 7022 in 2020. Gender-affirming mastectomy (GAM) is associated with improved psychosocial functioning and quality of life (QoL) among transgender and non-binary (TGNB) patients [1]. However, the perioperative period can be emotionally challenging, marked by concerns about surgical outcomes and physical discomfort. Effective communication between patients and providers during this time is crucial. It helps set expectations, ensures patient compliance, and reduces unnecessary utilization of health care services [2,3]. Several techniques have proven successful in patient education, including clinic teaching, written instructions, phone consultations, and bedside counseling [4,5]. However, while multiple opportunities exist for engaging with patients during inpatient procedures, outpatient surgeries, such as GAM, pose unique challenges due to limited face-to-face interactions between the patient and the care team [5]. Consequently, it is necessary for plastic surgeons specializing in gender-affirming care to optimize communication strategies to increase patient satisfaction.

The rise of patient-initiated communications (PIC) across surgical specialties has empowered patients to address unclear information or unanticipated concerns in the postoperative period. While these interactions can bolster a patient’s psychological well-being post-GAM, they can also lead to inefficiencies and increased burnout among surgeons and office staff [4,6]. To better perioperative education and reduce the frequency of PICs, it is critical to identify areas where current efforts do not meet patient needs. Given that TGNB individuals often experience heightened emotional and psychological distress related to gender dysphoria, effectively addressing these gaps can significantly influence their psychological well-being and satisfaction with surgical outcomes.

This study aims to (1) identify the prevalence and themes of PIC during the perioperative period among TGNB patients undergoing GAM and (2) examine the patient characteristics and surgical factors associated with these communications. The insights gained could enable gender-affirming care teams to develop targeted interventions that address the specific needs of TGNB patients, ultimately improving clinical outcomes and patient satisfaction. 

## 2. Methods

### 2.1. Study Design

Following Institutional Review Board approval, a single-institution retrospective review was conducted. The patient cohort comprised TGNB patients who underwent GAM between February 2018 to November 2022. All GAMs were performed by the senior author (G.A.D.). This study focused on PICs in the electronic medical records (EMRs) during the perioperative period, defined as 30 days before and after surgery. Each PIC, including phone calls and portal messages, was recorded in a separate note within the EMR by clinic staff, residents, or the primary surgeon. 

Data collected included patient characteristics, breast characteristics, perioperative details, and complications. Patient characteristics included age, race, body mass index (BMI), smoking status, diabetes mellitus, and duration of hormone use prior to surgery. Breast characteristics included cup size and degree of ptosis. Perioperative details included incision type (e.g., inframammary fold [IMF], keyhole, buttonhole), tissue resection weight, estimated blood loss, operative time, use of drains, and negative pressure wound therapy (NPWT) usage. The following complications were assessed: partial and total nipple graft loss (NGL), nipple discoloration, hematoma, seroma, wound infection, wound dehiscence, poor scarring, contour abnormalities, and reoperation. 

The primary outcome measured was the incidence of PICs within the defined perioperative period. The secondary outcomes included (1) reasons for PIC and (2) relationship between individual patient and operative characteristics and PIC frequency. 

### 2.2. Data Analysis 

We employed descriptive statistics to summarize the demographic and clinical characteristics of the study population. The Shapiro–Wilk test assessed the distribution normality of continuous variables. Categorical variables were reported as frequencies and percentages. Continuous variables were reported as means with standard deviations (SDs) or medians with interquartile ranges (IQRs), depending on their distribution. Pearson’s chi-squared (χ^2^) or Fisher’s exact tests (n < 5) were used for categorical variables, as appropriate. Mann–Whitney tests and unpaired two-tailed t-tests were used for continuous variables, as appropriate. All statistical analyses were performed using StataBE 17.0 (StatCorp LLC, College Station, TX, USA), with a *p*-value of <0.05 considered statistically significant.

## 3. Results

### 3.1. Patient Characteristics 

A total of 352 patients (704 breasts) were included for analysis. The median age was 25.0 (IQR: 9.0) years. The majority of patients self-identified as White (n = 238, 67.6%), followed by Black (n = 81, 23.0%). Median BMI was 28.5 (IQR: 8.5) kg/m^2^. Median CCI was 0.0 (IQR: 0.0). Median driving distance to the hospital was 31.4 (IQR: 49.0) miles. Median ZIP code-based income was USD 83,190 (IQR: USD 50,229). Median duration of hormone therapy before surgery was 23.0 (IQR: 22.0) months. A total of 119 (33.8%) patients had a history of major depressive disorder (MDD) or generalized anxiety disorder (GAD). Further patient characteristics are summarized in Table 1.

### 3.2. Perioperative Details and Outcomes

The majority of breasts had a preoperative B cup size (n = 200, 28.4%), followed by C (n = 178, 25.3%), D (n = 146, 20.7%), ≥DD (n = 40, 5.7%), and A (n = 11, 3.1%). The majority of breasts had grade III ptosis (n = 192, 27.3%), followed by grade II ptosis (n = 118, 16.8%), grade I ptosis (n = 80, 11.4%), and no ptosis (n = 162, 23.0%). The most frequently used incision type was along the IMF (n = 331, 94.0%). Median tissue resection weight was 1124 g (IQR: 1177). Median operative time was 90.0 (IQR: 33.0) minutes. Postoperatively, 335 (95.2%) patients utilized a drain, with a median time to drain removal of 8.0 (IQR: 4.0) days. One hundred eighty-four (52.3%) patients received NPWT postoperatively, with a median time to NPWT removal of 7.0 (IQR: 3.0) days.

By a median follow-up duration of 9.6 (IQR: 4.2) months, the most frequent complications were partial FNG loss (n = 26, 7.4%), followed by nipple discoloration (n = 23, 6.5%), hematoma (n = 17, 4.8%), total FNG loss (n = 9, 2.6%), seroma (n = 8, 2.3%), wound dehiscence (n = 6, 1.7%), and surgical site infection (SSI; n = 6, 1.7%). 

### 3.3. Patient-Initiated Communication

A total of 285 (74.6%) patients engaged in PIC, resulting in 659 unique PICs. Of these, 215 (32.6%) were initiated in the preoperative period, and 444 (67.4%) were initiated in the postoperative period. Mean number of PICs was 0.7 ± 1.3 preoperatively and 1.3 ± 1.7 postoperatively (*p* < 0.001). 

**Figure 1:**
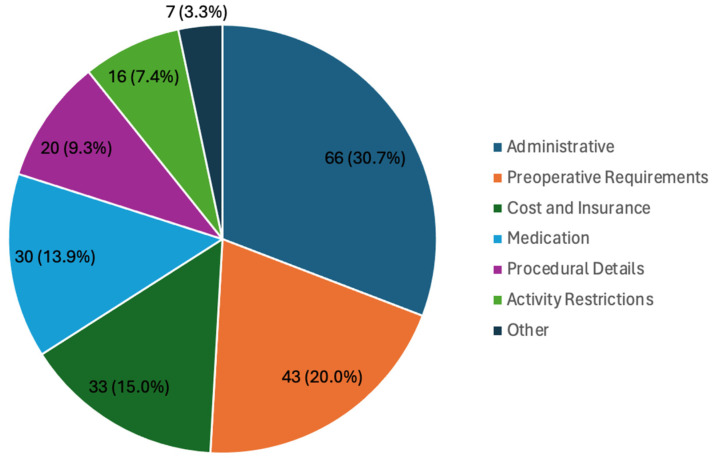
Preoperative patient-initiated communications.

**Figure 2:**
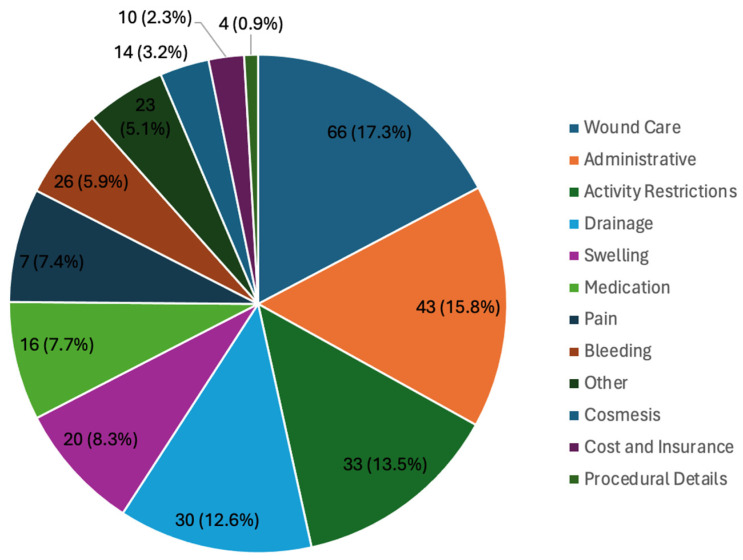
Postoperative patient-initiated communications.

Preoperatively, PICs addressed concerns such as administrative issues (AI; n= 66, 30.7%), preoperative requirements (n = 30, 20.0%), and cost and insurance (n = 33, 15.0%). AIs included questions or concerns about scheduling (n = 41), returning to work or taking a leave of absence (n = 19), COVID-19 (n = 6), and free nipple grafting (FNG; n = 5). The most common medication-related questions were about discontinuing medications such as testosterone injections (n = 20) and managing pain (n = 8; Figure 1). Postoperatively, PICs addressed wound care (n = 77, 17.3%), AIs (n = 70, 15.0%), activity restrictions (n = 60, 13.5%), drainage concerns (n = 56, 12.6%), and swelling (n = 37, 8.3%). AIs included questions or concerns about returning to work or taking a leave of absence (n = 55) and scheduling (n = 40). The most common medication-related inquiries concerned pain management (n = 18), side effects (n = 7), and medication refill requests (n = 6). The most common wound care-related questions addressed concerns about bandages (n = 22), binders (n = 16), appearance (n = 11), and NPWT (n = 8; Figure 2).

### 3.4. Univariate Analyses

Older patients engaged in higher levels of PIC compared to younger patients (β = 0.234, *p* = 0.001). Patients with a history of MDD or GAD engaged in higher levels of PIC than those without such psychiatric diagnoses (2.4 ± 3.0 vs. 1.7 ± 1.9, respectively; *p* = 0.019). Patients who did not have postoperative drains were more likely to engage in PIC than those with drains (n = 16/17, 94.1% vs. n = 236/334, 70.7%, respectively; *p* = 0.025). There were no other significant associations found between patient characteristics, perioperative details, or outcomes and the frequency of PIC. 

## 4. Discussion

GAM is a critical step in the gender affirmation process for TGNB individuals, significantly improving mental health, body satisfaction, and overall QoL [1]. The perioperative period in gender-affirming care presents unique challenges and opportunities for communication between patients and healthcare providers. This study explores the factors influencing the frequency and nature of PICs in the perioperative period of GAMs. This is particularly crucial for TGNB patients who face unique challenges and higher rates of anxiety and depression compared to the general population. In our study of 352 patients, 74.6% initiated communication, resulting in 659 unique PICs. We also found that age, psychiatric diagnosis, and use of postoperative drains were key predictors of PIC frequency. These findings are crucial for developing targeted interventions that address the specific needs of this population.

In our study, older patients demonstrated a higher likelihood of initiating communication with their surgeon during the perioperative period, a trend supported by the existing literature [7,8]. Older adults tend to have greater health literacy and confidence in navigating the healthcare system, often developing long-term relationships with providers [9,10,11]. Conversely, younger adults, particularly those aged 18 to 34, tend to rely more on online sources and are less inclined to initiate direct communication [12,13]. They also exhibit lower trust in physicians, especially concerning confidentiality—a critical concern in gender-affirming surgery [14,15]. Safer et al. reported that TGNB individuals felt less respected by providers who did not honor their gender identity, displayed negative attitudes, feared legal consequences, lacked coordinated care, struggled with payment frameworks, or showed insensitivity to broader social barriers to care [16,17,18]. Altogether, understanding these age-related differences is crucial for tailoring communication strategies and ensuring effective patient-provider interactions across all age groups.

It is important to acknowledge the high incidence of GAD (29.6%) and MDD (33.3%) among this demographic, compared to the cisgender population (19.1% and 18.4%, respectively) [19,20,21,22,23]. In our cohort, 33.5% of patients had a history of GAD or MDD. Additionally, these patients were significantly more likely to initiate communication with their surgeon in the perioperative compared to those without such psychiatric histories. This finding has several implications. First, existing literature highlights a significant relationship between psychiatric diagnoses and increased PIC, particularly in the preoperative phase, as individuals seek clarification and reassurance to alleviate their anxieties [7,24,25,26]. Moreover, patient anxiety may also encompass concerns about postoperative healing, procedural details, and medication management, contributing to the higher frequency of phone calls and messages. Not all patients in our study underwent comprehensive evaluations for a history of GAD or MDD, which could introduce bias that might affect our findings. Nonetheless, proactively identifying these patients during consultations and preoperative visits could be a valuable strategy for addressing their concerns in advance.

Given that “top surgery” is often the initial gender-affirming procedure for this patient population, unfamiliarity with the gender-affirming surgical process is common [27]. In our study, AIs were the predominant reason for PIC preoperatively. These inquiries typically included requests to proceed with surgery, changes to scheduling, and questions about insurance authorization. Patients considering GAM may prefer to plan their surgery over the phone, allowing them to consult with their support systems, find an appropriate time frame, and address financial concerns, given that insurance may not always cover GAMs. These personal deliberations are vital, as they help patients build trust with their surgeon and engage in patient-centered care. 

Postoperatively, patient concerns included insurance authorization, wound care, binder placement, activity restrictions, drainage, and swelling. There were also significant communications about medications, specifically clarifications on usage, requests for specific pharmacy deliveries, and refill requests. Additionally, pain management was a notable area of concern, with patients frequently inquiring about appropriate pain medications and refill protocols. These topics are consistent with findings from other research on patient concerns. For instance, in Mohs surgery, common reasons for PIC included wound care, infection, bleeding, and pain [7,8]. In orthopedic trauma settings, the rationales for PICs included pain control (22%), wound care (16%), and questions about discharge medications (8%) [28]. In our study, patients without drains engaged in higher levels of PIC, likely due to uncertainty during their postoperative recovery regarding measurable healing indicators, such as drain fluid collections. Moreover, the absence of drains might encourage patients to be more proactive in their recovery, leading them to communicate more frequently to seek advice on self-care and symptom management. 

The use of visual aids and graphic tools has been shown to enhance patient understanding before various medical procedures [29,30]. Callery et al. found that combining verbal and written education with access to online resources for breast augmentation surgeries reduced patient phone calls, increased patient satisfaction, and improved documentation [31]. Based on these findings, plastic surgeons should consider implementing such tools and dedicating more time in the preoperative period to clearly outline expectations, potential complications, and medication protocols. In addition to perioperative education, it is crucial for plastic surgeons to deliver sensitive and individualized care, using gender-neutral language, appropriate personal pronouns, and conducting sensitive physical exams. Furthermore, it is essential to show empathy towards patients who may not ask many questions preoperatively, but experience distress postoperatively. For example, outpatient departments can encourage patients to prepare written questions before their face-to-face visits. This practice not only aids in maintaining privacy, but also ensures that all patient concerns are addressed. By fostering an environment that encourages open communication, care providers can better support these patients and potentially reduce the frequency of PICs. These practices are crucial for improving the patient experience and fostering a foundation of trust and respect [32,33].

In our practice, we dedicate significant time and resources to developing a comprehensive strategy that addresses preoperative anxieties, clarifies procedural details, and sets clear postoperative expectations regarding outcomes and recovery. This approach is based on evidence that highlights the critical role of trust in patient–provider relationships [34,35,36]. It also adheres to the World Professional Association of Transgender Health (WPATH) Standards of Care (SOC), which emphasize the importance of effective communication in surgical interventions [37,38]. The SOC advocate for face-to-face consultations to discuss surgical techniques, risks, and expected outcomes. These standards further stress the importance of providing comprehensive information in a language the patient understands [37]. Furthermore, our institution advocates for continuous multidisciplinary communication with social workers, mental health providers, and LGBTQ+ community leaders to provide robust support for patients throughout the perioperative period.

### Limitations

The single-institution, retrospective nature of this study may limit the generalizability of our findings to broader populations. Additionally, our findings are confined to the data documented in the EMRs. It is possible that some PICs were not appropriately recorded, and thus, were omitted from our analysis. Nonetheless, our study identifies general trends and themes in patient characteristics that may predispose to increased PIC in gender-affirming surgeries. Future research is warranted to evaluate the impact of implementing patient education interventions on the frequency of PIC during the perioperative period.

## 5. Conclusions

Perioperative PIC occurs among most GAM patients at our institution. In our study, age, psychiatric diagnosis, and postoperative drain use were significant predictors of PIC in TGNB patients undergoing GAM. Ensuring sufficient support staffing and providing clear postoperative instructions about activity restrictions, drainage, and expected complications can effectively reduce the incidence of postoperative PIC in high-volume centers and enhance overall patient satisfaction rates.

## Figures and Tables

**Table 1:** Patient characteristics.

	Total (%)
	352 (100.0)
**Demographics**	
Age (years); Median (IQR)	25.0 (9.0)
Race	
Black	81 (23.0)
White	238 (67.6)
Hispanic	2 (0.6)
Asian	12 (3.4)
Other	19 (5.4)
BMI (kg/m^2^); Median (IQR)	28.5 (8.5)
Driving Distance to Hospital (miles); Median (IQR)	31.4 (49.0)
ZIP Code-Based Income (USD); Median (IQR)	83,190 (50,229)
**Comorbidities**	
CCI; Median (IQR)	0.0 (0.0)
DM	9 (2.6)
Psychiatric History *	119 (33.8)
MDD	85 (24.1)
GAD	108 (30.7)
Smoking Status	
Current	32 (9.1)
Former	51 (14.5)

Abbreviations: IQR: interquartile range; *: psychiatric diagnosis included history of MDD or GAD only; MDD: major depressive disorder; GAD: generalized anxiety disorder.

## Data Availability

The datasets analyzed during the current study are available from the corresponding author on reasonable request.
